# Expanding the Vision for Differentiated Service Delivery: A Call for More Inclusive and Truly Patient-Centered Care for People Living With HIV

**DOI:** 10.1097/QAI.0000000000002549

**Published:** 2020-11-23

**Authors:** Peter Ehrenkranz, Anna Grimsrud, Charles B. Holmes, Peter Preko, Miriam Rabkin

**Affiliations:** a Global Health, Bill & Melinda Gates Foundation, Seattle, WA;; b HIV Programmes & Advocacy, International AIDS Society, Cape Town, South Africa;; c Center for Innovation in Global Health, Georgetown University, Washington, DC;; d ICAP at Columbia University, New York, NY.

**Keywords:** HIV, ART, differentiated service delivery, Universal Health Care, noncommunicable diseases, family planning, tuberculosis preventive therapy, sustainability

## Abstract

Simplifying antiretroviral therapy for clinically stable people living with HIV (PLHIV) is important but insufficient to meet their health care needs, including prevention and treatment of tuberculosis and noncommunicable diseases, routine primary care, and family planning. Integrating these services into differentiated service delivery (DSD) platforms is a promising avenue to achieve such coverage. We propose a transition from an HIV-focused “DSD 1.0” to a patient-centered “DSD 2.0” that is inclusive of additional chronic care services for PLHIV.

**Discussion:**

The lack of coordination between HIV programs and these critical services puts a burden on both PLHIV and health systems. For individual patients, fractionated services increase cost and time, diminish the actual and perceived quality of care, and increase the risk that they will disengage from health care altogether. The burden on the health system is one of inefficiency and suboptimal outcomes resulting from the parallel systems required to manage multiple vertical programs.

**Conclusions:**

DSD 2.0 provides an opportunity for the HIV and Universal Health Coverage agendas—which can seem to be at odds—to achieve greater collective impact for patients and health systems by integrating strong vertical HIV, tuberculosis and family planning programs, and relatively weaker noncommunicable disease programs. Increasing coordination of care for PLHIV will increase the likelihood of achieving and sustaining UNAIDS′ goals of retention on antiretroviral therapy and viral suppression. Eventually, this shift to DSD 2.0 for PLHIV could evolve to a more person-centered vision of chronic care services that would also serve the general population.

## INTRODUCTION

Differentiated service delivery (DSD)—a “patient-centered approach that simplifies and adapts HIV services across the cascade to serve the needs of people living with HIV (PLHIV) better and reduce unnecessary burdens on the health system^1^”— has emerged as a core tenet of HIV programs in resource-limited settings.^2–4^ Practically speaking, DSD is operationalized by adjusting the frequency of visits, the location of service delivery, the cadre of health care worker, and the package of services according to the needs of different groups of PLHIV. Varying the configuration of these factors results in DSD treatment models that often separate clinical visits from antiretroviral therapy (ART) refills including “fast track” refill pick-ups in the facility or community, client-managed community adherence groups and health care worker managed groups.^5^

Efforts are ongoing to define the extent and impact of DSD scale-up in sub-Saharan Africa.^6,7^ Current data are sufficient to show that the numbers of people receiving extended medication refills, which is one component of DSD, has risen dramatically. A recent analysis of US President's Emergency Plan for AIDS Relief (PEPFAR)-supported countries (excluding South Africa) reported an increase in the percentage of patients receiving 3 or more months of refills from 46% to 69% between October 2019 and June 2020.^8^ The COVID-19 epidemic accelerated this rapid change in dispensing practices and the uptake and adaptation of various DSD models across sub-Saharan Africa.^9^

Preliminary data evaluating the outcomes of DSD treatment programs across multiple countries^10^ support the concept that continued scale-up of DSD is critical to attaining the ambitious coverage, retention, and quality targets of the global HIV response.^11^ However, simplifying ART delivery for clinically stable PLHIV is not sufficient to meet all of their health care needs, which often include prevention and treatment of tuberculosis (TB), routine preventive and primary care, family planning (FP), and noncommunicable disease (NCD) services. Providing for these comprehensive needs is essential to the goals of the growing movement to implement Universal Health Care (UHC),^12^ and integrating some of these services into DSD platforms is a promising avenue to achieve such coverage.^13^

In this commentary, we highlight opportunities to address the unmet need for coordination of HIV and other priority services and propose a transition from an HIV-focused “DSD 1.0” to a truly patient-centered “DSD 2.0” that is inclusive of additional chronic care services. An initial focus could be on PLHIV, but DSD 2.0 provides a platform for offering any longitudinal or chronic care service to the general population. The goal would remain the same: to scale-up an evidence-based patient-centered approach that reduces unnecessary burdens for individuals and the health system. The proposed DSD framework thus provides an opportunity for the HIV and UHC agendas—which can seem to be at odds at first glance—to leverage each other and achieve greater collective impact.^14^

### A Call for DSD 2.0

UNAIDS has set aspirational goals to end the AIDS epidemic by 2030.^11^ Global stakeholders are further proposing plans to sustain HIV epidemic control, but the definition of sustainability and the roadmap for achieving it remain unresolved.^2,15^ It is clear that the historically vertical approach to HIV programming does not naturally lend itself to sustainability in a world whose attention is rapidly shifting toward UHC goals. This shift away from vertical programming is likely to be accelerated by the COVID-19 pandemic, which has emphasized the importance of resilient and integrated national health systems.^16^

DSD 1.0's intentionally narrow focus on simplifying HIV services and reducing unnecessary burdens on the health care system was critical for building consensus, establishing an evidence base, and driving efforts to achieve scale. However, it does not enhance access to the full range of prevention and treatment services needed by recipients of care. Strategically addressing these needs for the long-term will be critical to maintaining patients' experience of quality of care and maximizing retention,^17^ and to ensuring both HIV viral suppression and healthy aging for PLHIV.^18^ This expanded focus is the essence of what we call “DSD 2.0.”

WHO's 2016 HIV guidelines provide a template for DSD 2.0. They emphasize the importance of integrating ART services with the most common vertical programs that require repeated follow-up: TB prevention and treatment, FP, and chronic NCDs.^4^ A majority of adults with HIV will require longitudinal care for such non-HIV services. All PLHIV are eligible for a course of TB preventive therapy (TPT) and between 5% and 10% will develop TB at some point in their lifetimes.^19^ The unmet need for contraception among women on ART is well-documented, approaching 50% in some settings.^20^ Chronic NCDs such as diabetes, hypertension, and mental health disorders are also a major issue. Among adults on ART in South Africa, more than 15% have hypertension, more than 5% have diabetes,^21^ and, as a recent review emphasized, there is a real risk that poor quality of care for such NCDs may undermine the investments made to strengthen HIV programs.^22^

In most sub-Saharan African countries, PLHIV requiring care for any of these other health needs (with the intermittent exception of TB services) usually seek them in physical locations other than the HIV clinic and often on a different appointment schedule. FP programs are usually run vertically. Clinicians providing HIV services are not always trained in provision of FP nor do they routinely have the commodities available to provide FP at the point of HIV care.^23^ Women living with HIV are routinely referred to FP clinics, but there is often no communication between the 2 programs to align appointments or to determine best approaches to managing potential interactions between ART and contraceptives.^24^ The situation is worse for integration with chronic NCD programs. Some countries with strong HIV programs do not have updated national guidelines for hypertension or diabetes, let alone consistent supplies of medications outside of referral facilities. Although HIV commodities are prioritized and often paid for by external donors, NCD commodities must be borne by the country and, as a result, are often accompanied by user fees. Where NCD guidelines and medications are available, there is likely little alignment of appointment scheduling or refills with the ART program,^25,26^ let alone the provision of NCD screening and treatment within HIV programs. The lack of coordination between these critical services and HIV care puts an unnecessary and, to date, unquantified burden on both the patients and the health system.

The burden on patients is experienced in 2 ways—once because a concurrent condition like hypertension often goes undiagnosed in any resource-poor setting and again because, once diagnosed, treatment services are only available in a parallel setting. For example, a patient with both HIV and hypertension may only need to visit an HIV clinic twice a year, but could be required to visit a different clinic monthly for hypertension care. The patient may also be required to pay out-of-pocket for the hypertension services. This fragmented care may prove to be frustrating for many patients and actually disincentivize them to remain engaged in any health services. If DSD remains narrowly focused on ART, it may make HIV treatment more convenient and efficient, but will not comprehensively address the health needs of people engaged in HIV treatment. This outcome may diminish the actual and perceived quality of care, and push patients closer to a “tipping point” where they choose to disengage from the health system altogether, leading to worse outcomes for both conditions.^27^

The burden on the health system results from having to maintain parallel systems to manage HIV, FP, hypertension, diabetes, and other chronic NCD programs. For example, HIV medical record systems may be robust, but few of them contain information about need for or use of other health services by PLHIV, limiting their usefulness for quality improvement or planning. Meanwhile, few countries have longitudinal record keeping for non-HIV services at all. This blind spot is critical, and reflects similar inefficiencies in supply chain, laboratory, human resources, and other key health system building blocks.^28^

### The Path to DSD 2.0

Similar proposals for greater integration of services have been made previously.^13^ Although there has been some success with coordinating TB/HIV services in particular settings,^29^ integration of other services has faced barriers to scale, including lack of resources to pay for the non-HIV services and accompanying lack of centralized support to establish a functioning large-scale public-sector program for conditions such as diabetes or hypertension.^30^ With the concerns about decreasing international financial support for the HIV response and the accelerating global movement toward UHC,^31^ there have been multiple calls for policies, plans, and resources to facilitate integration, including health insurance programs and civil society engagement.^32^ However, there is little attention given to the need for defining practical common ground between the UHC and HIV movements, which, if addressed, could provide momentum to overcoming remaining logistical and resource challenges.^14^

DSD could be that common ground. The process of bringing DSD 1.0 for ART delivery to scale has defined a pathway for providing patient-centered treatment models for longitudinal care. DSD 1.0 has adapted the public health approach to HIV in a way that minimizes unnecessary demands on a constrained health system and is more responsive to the priorities of a given patient and community—who, in turn, are trusted to assume greater responsibility for self and/or peer management. Engaging local stakeholders to determine which additional services to include in a country-specific version of DSD 2.0 will enable consideration of community priorities, local health system strengths, resources, and short and long-term objectives for comprehensive care.

For example, to meet DSD 1.0 goals of easing access to medication refills, South Africa's HIV program developed innovative channels for prepacking routine medication for HIV, hypertension, and diabetes. This was enabled by a national approach to support adherence to treatment for HIV, TB, and chronic NCDs—as opposed to a vertical HIV program.^33^ The Central Chronic Medicines Dispensing and Distribution (CCMDD) program described in the national guidelines is a harbinger of South Africa's plan toward National Health Insurance and UHC. Although antiretrovirals make up most medicines currently packaged by the CCMDD platform, non-HIV medicines have also been included with little additional marginal cost. Of the almost 2.1 M patients enrolled in the CCMDD program as of February 2020, 64% were receiving ART alone, 23% were receiving only medicines for chronic NCDs, and 13% were receiving both ART and medicines for chronic NCDs.^34^ The program has been shown to reduce stockouts of essential medicines in South Africa,^35^ and future studies are expected to evaluate whether such more consistent supply leads to improved HIV and chronic NCD outcomes. In the meantime, Uganda is exploring adapting a similar model.^34^

Many countries have made national commitments to a broad range of health systems reforms aimed at improving health outcomes. In some cases, these advances have included health financing proposals that could pay for the implementation of large-scale integrated public sector programs that serve multiple health care needs.^36^ Kenya is well-positioned to lead the continent in achieving these aims; however, its current UHC plans are not clear on what aspects of HIV care are included and civil society has questioned if, as a vertical disease program with external funders, HIV services are expected to continue to be implemented in parallel.^37,38^ Collaborative development of a DSD 2.0 strategy could be a first step that will bring these UHC and HIV programs together to support people needing any longitudinal care. Funded appropriately and implemented well, the results could include improvements to patients' experience of care, better individual and public health outcomes, and increased efficiency for the health care system. Such integration could provide needed momentum for governments, advocates, and donors to recognize the opportunity of leveraging the strengths of historically vertical programs. Critically, evidence from models of integrated NCD/HIV services suggests that the effort could be cost-effective, and evidence from pilots has suggested that the marginal costs per HIV patient would be low (<$7/PLHIV/year).^39,40^

### Building From TB, FP, and NCDs to UHC

The UHC Political Declaration made at the 2019 United Nations General Assembly specifically called for “integrated quality health services” that are “people-centred.^41^” UHC2030's action agenda includes the call to “put better quality on par with expanded coverage… this requires new investment and delivery models, and collaborations through participatory and inclusive service delivery redesign and planning.^42^” The UNGA declaration and UHC 2030 movement are providing the political commitment, attention, and funding from countries and donors, and use of consensus indicators to track progress. The concept of DSD 2.0 provides an entry point to unlock synergies between HIV programming and a UHC-oriented service delivery redesign.

We suggest an initial emphasis on TPT and TB treatment for PLHIV. TPT is well along the way to integration with HIV programs at scale and is already delivered at full scale in Kenya.^29,43,44^ Ongoing pilots in Zambia and Zimbabwe are assessing the feasibility and acceptability of TPT in the context of less-intensive DSD models, specifically Fast Track and Community ART Refill Groups, to both patients and health care workers.^45,46^ To assist with further scale-up, the International AIDS Society recently published guidance on how TPT may be integrated into differentiated ART delivery models for clinically stable clients.^47^ Similarly, there is an opportunity for TB treatment to be integrated into DSD programming for PLHIV. At least 11 countries in sub-Saharan Africa permit multi-month dispensing of TB treatment, including Cameroon, Ethiopia, Kenya, Nigeria, South Africa, Uganda, and others.^48,49^

We propose FP for women living with HIV as a second priority.^50^ It is an unmet need across Africa, and women living with HIV who manage their own ART may be a receptive audience for multi-month refills of oral contraceptives or longer acting self-administered contraceptives, such as Sayana Press,^51^ a self-injectable medroxyprogesterone acetate. There are limited published examples of FP being offered to PLHIV as part of a DSD model, but a recent presentation described how ART community adherence groups in Kenya aligned their ART pickup with distribution of implants, oral pills, and injectables.^52^ We anticipate that there are many other innovations in delivery of FP to women living with HIV and are seeking to define and share them.

Third, we propose that integration efforts focus on chronic NCDs such as hypertension and diabetes among PLHIV. Availability of treatment for these conditions lags behind TB and FP. However, these NCDs will become an ever more urgent issue as PLHIV age. Like HIV treatment, successful NCD treatment requires behavior change, (often) long-term medication use, and lifelong clinical and laboratory monitoring.^53^ Therefore, like PLHIV who are clinically stable on ART, individuals who are stable on hypertension and diabetes treatment may be good candidates for appointment spacing, multi-month prescribing, and community-based services. Preliminary data from Eswatini, for example, show the successful implementation of DSD for people with both HIV and NCDs via a less-intensive DSD “club” model.^54^

Importantly, similar to HIV treatment, all of these services—TB, FP, NCDs—can be managed by nonphysician clinicians or patients themselves. For routine cases, the availability of streamlined management and referral algorithms enables the standardized procurement, training, and workforce support needed for decentralized services, including community-based service delivery. In addition, neither symptom monitoring nor simple diagnostic testing requires a trained health worker. Self-collection of dried blood spots from PLHIV to determine viral load suppression and eligibility for a refill of ART without a clinical consultation is already being tested in South Africa.^55^ Similarly, self-monitoring of blood pressure by PLHIV with hypertension has been shown to be acceptable and feasible in Eswatini.^56^ Remote testing of HbA1C for monitoring of diabetes management and determination of eligibility for a refill of oral therapy could occur in similar fashion. In turn, the availability of self-monitoring will enable the use of multi-month prescriptions and dispensing.

In places where DSD services for HIV are only now emerging and NCD services are limited, as in much of West and Central Africa, there is an opportunity for a paradigm shift. Many of these countries are at early stages of providing routine care for patients with any chronic health need—HIV, FP, hypertension, diabetes, or even mental health.^57^ If they could develop an integrated chronic disease DSD program from the start, it could leapfrog over DSD 1.0 to minimize the intensity of care for PLHIV, likely improve HIV outcomes and ideally be supported by UHC planning from the outset.

## CONCLUSION

**FIGURE 1.**
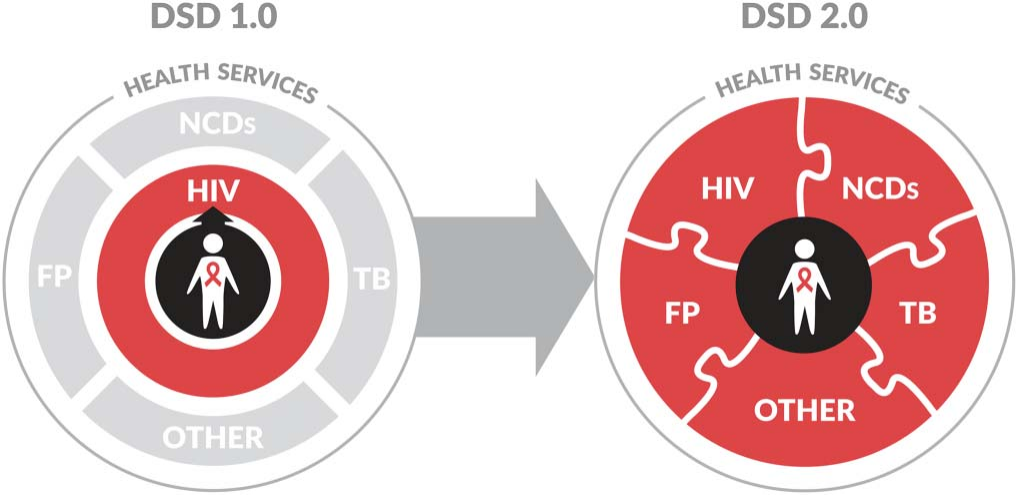
A description of the proposed transition from an HIV-focused “DSD 1.0” to a patient-centered “DSD 2.0.” DSD 2.0 is inclusive of additional chronic care services for PLHIV, such as TB, FP, NCDs, and others.

The proposed transition from “DSD 1.0” to “DSD 2.0” recognizes the success of HIV programs in providing patient-centered programs for chronic HIV care and pushes them to do more to provide a comprehensive and integrated package of high-quality health services to their patients [Fig. 1]. It also challenges the UHC response, largely centered around provision of quality primary health care (PHC), to learn from and assist in the provision of broader care to those living with HIV. The costs and benefits of this comprehensive approach need to continue to be tested with modeling and empirical studies, including those cited in a recently published research agenda,^58^ but its strength is that it recognizes that the needs of PLHIV will change over time and are not limited to HIV care alone. The more PLHIV's touch points are coordinated with the health system, the more likely the goals of retention and viral suppression can be achieved and sustained. In parallel, this evolution provides a tangible path for integration between heretofore strong vertical HIV programs, other vertical programs such as TB and FP, and relatively weaker programs for PHC addressing chronic NCDs. In the future, this shift to DSD 2.0 for PLHIV could evolve to a more person-centered vision of chronic care services and universal health care for all—a to-be-defined future state that is paid for through a combination of national health insurance schemes and donor funding.

If a health system can provide hypertension drugs and ART in a manner that decreases the need for frequent health facility visits—for example, by providing multi-month refills and/or offering refills at private pharmacies—a next step would be to provide that service for someone whose needs are limited to treatment for hypertension. In this way, the health systems that underlie both UHC and HIV will be strengthened by building the capacity to support multiple patient needs simultaneously rather than prioritizing historically vertical programs.
